# TikTok war humor: social and psychological functions of humor videos by micro-influencers and ordinary users during conflict

**DOI:** 10.3389/fpsyg.2025.1637194

**Published:** 2025-08-25

**Authors:** Nili Steinfeld, Hananel Rosenberg, Michal Mahat-Shamir

**Affiliations:** ^1^School of Communications, Ariel University, Ariel, Israel; ^2^Department of Social Work, Ariel University, Ariel, Israel

**Keywords:** TikTok, humor, social media content, conflict, war, resilience, media psychology, well-being

## Abstract

While much research highlights the negative psychological impacts of social media use, the current study examines how, in the aftermath of collective trauma, social media platforms can serve as spaces for resilience-building through the creative use of humor. On October 7, 2023, Hamas launched a large-scale surprise attack on Israel from the Gaza Strip, resulting in over 1,200 Israeli casualties, 253 hostages, and extensive damage. Recognized as the deadliest terrorist attack in Israel’s history, it led to a war, causing significant distress and trauma among all the peoples of the region. Surprisingly, just days after the attack, humorous videos emerged on TikTok, uploaded by Israeli influencers, citizens, and reservists. This study examines the interaction between humor, TikTok performance, and public trauma during war. A quantitative content analysis of 257 TikTok videos from the initial post-October 7th period identified the styles, characteristics, and psychological functions of humor, and the unique nature of TikTok humor. Additionally, a thematic analysis of in-depth interviews with seven Israeli TikTok influencers explored their motivations for creating and sharing humorous content during this period and the influence of audience interactions on their actions. Findings show that humorous content followed platform rules, with trends, TikTok formats, and parodies being dominant. Creators aimed to boost morale while avoiding controversial political topics. Civilians and soldiers used humor differently: civilians focused on coping and stress at home, while soldiers focused on frontline and army routine. Interviews revealed TikTok as a therapeutic space, with humor serving as a defense mechanism. The creative process was calculated, filled with uncertainties and heightened sensitivity, as creators and users practiced resilience-building during their darkest hours.

## 1 Introduction

Over the past decades, Internet use has expanded rapidly across all age groups, with over four billion users worldwide as of 2021 (Internet World Stats, 2021). This surge has been accompanied by growing concerns regarding problematic internet and social media use, characterized by poor time management, obsessive thinking, mood regulation difficulties, social and professional impairment, and relapse tendencies (Stănculescu and Griffiths, 2022). Studies estimate that the prevalence of problematic use ranges from 20.0 to 44.6% across different global regions (Endomba et al., 2022), with documented associations to mental health challenges such as anxiety, loneliness, decreased subjective well-being, and depression (Kagan et al., 2025; McCrae et al., 2017; Shan et al., 2021). While much attention has been devoted to these adverse outcomes, social media platforms can also function as important spaces for resilience-building, particularly when used for humor-based content. Research indicates that humor serves as a vital coping mechanism in both primary and secondary trauma, promoting well-being and post-traumatic growth (Craun and Bourke, 2014; Cofini et al., 2014). Building on these insights, the current study explores the styles and functions of humor appearing on social media following the collective traumatic event of the October 7 attack on Israel and examines how platform-specific features shape their expression and meaning.

## 2 Literature review

### 2.1 The October 7th attacks

On Saturday, October 7, 2023, Hamas (The Islamic Resistance Movement, the governing body in the Gaza Strip) launched a large-scale surprise attack from the Gaza Strip on Israel. The organization simultaneously unleashed massive rocket fire at southern communities while hundreds of its terrorists infiltrated the confines of the Gaza Strip and attacked military bases, residential communities, and the Nova music festival, attended by thousands of young participants. Terrorists invaded homes in villages near the border, set them on fire, and murdered entire families. Over 1,200 Israelis were killed and 253 civilians and soldiers were taken hostage (Ministry of Foreign Affairs, 2023; Pitcho, 2024). On the following day, the Lebanese militant group Hezbollah launched rocket fire at northern Israeli communities, forcing many residents of the North, in addition to the residents of communities around Gaza, to evacuate their homes. The October 7th attack is widely regarded as the deadliest terrorist attack in the history of the State of Israel and the most devastating disaster to befall the Jewish people since the Holocaust (Lahav and Ben-Ezra, 2024). During that Saturday, and in the days that followed, hundreds of thousands of citizens were drafted into military reserve. The war that ensued between the State of Israel and its neighboring entities in the aftermath of these events continues, at the time of writing this paper,^1^ to exact a substantial toll on both sides, reflected in significant physical casualties, injuries, and adverse effects on psychological well-being.

Public response to the attack included strong feelings of distress, anxiety, loss of control, and individual and collective trauma (Feingold et al., 2024; Levi-Belz et al., 2024). It is estimated that over 30% of the Jewish population experienced symptoms of post-traumatic stress in the months following the events (Yamin, 2023). Among the factors that intensified these experiences was the extensive visual documentation that accompanied the terrorist attack, made and uploaded to the Internet by Hamas terrorists during and after the attack, in addition to videos from security cameras, dashboard cameras, and the smartphones of victims (Yamin, 2023).

Surprisingly, just a few days after the attack, humorous videos began to appear on TikTok, uploaded by well-known Israeli social media influencers, ordinary citizens, and reservists. They included references to Israel’s heightened atmosphere of anxiety, disappointment with the response of international organizations that refused to condemn Hamas’ actions, and the sharing of experiences from the unique camaraderie and dynamics of public and military conscription during this period. These videos became popular, and several lesser-known TikTok creators became social media influencers. While the use of humor in situations of anxiety, during traumatic times such as war, is a well-documented phenomenon (Yehorova et al., 2023), the public nature of sharing humorous videos on a social platform during such an emotionally charged period and so soon after traumatic events is striking. TikTok enables the distribution of content to a wide range of audiences, domestic and international, but its unique affordances also invite users to create personal and revealing content. This study addresses this tension and provides a unique perspective on the interaction between humor, its performance on TikTok, and situations of public trauma and war. We combine quantitative content analysis of TikTok videos from the initial post-October 7 period with a thematic analysis of in-depth interviews with influencers, to map and define the unique nature of humorous performances on TikTok and their psychological functions, as well as understand influencers’ motivations for producing and publishing humorous content during this period and how their interactions with audiences shaped their actions.

### 2.2 The role of humor in public and online spaces during anxiety, trauma, and war

Humor is widely recognized as a coping mechanism for managing anxiety, trauma, and distress in contexts such as wars, disasters, and collective traumatic events. Anxiety refers to emotional states characterized by heightened worry, tension, and physiological arousal in response to perceived threats (Spielberger and Reheiser, 2009). Trauma involves intense psychological responses to overwhelmingly distressing events that disrupt normal coping capacities (Herman, 2015). Trauma can manifest as primary trauma (direct exposure to traumatic events), secondary trauma (indirect exposure through media or interpersonal relationships), or public trauma (collective exposure experienced by societies or communities during shared crises) (Hirschberger, 2018; Silver et al., 2021). Post-trauma refers to ongoing psychological consequences following traumatic exposure, potentially resulting in long-term anxiety, post-traumatic stress, or resilience and post-traumatic growth (Tedeschi and Calhoun, 2004). Although traumatic events themselves are not humorous, the perception of humor as a mechanism of sublimation (Freud, 1928) leads to its adoption in these situations as an adaptive defense mechanism for healthy and mature coping (Vaillant, 2000). Indeed, self-enhancing humor as a coping mechanism has been found to have a profound therapeutic effect (Yim, 2016), which helps reduce psychological suffering, anxiety, and stress (Mahat-Shamir and Kagan, 2022). Humor use correlates strongly with mental well-being (Karakuş et al., 2014), higher levels of perceived social support (Cann et al., 2010), a more optimistic view of the future (Hackney, 2011), and mental stamina in challenging situations (Pande, 2014).

In situations of stress and anxiety, humor manifests in various styles: aggressive humor and mockery directed at the threat (Stokker, 1991; Martin, 2007) help release pent-up tension within a person (Cheng et al., 2019) and maintain mental stability under unbearable conditions (Ford and Spaulding, 1973). Self-deprecating humor provides a person with a reflective perspective on their difficulties and allows for a new interpretation of threatening situations (Ostrower, 2015). Dark humor, also known as gallows humor, has been recognized as a tool for alleviating psychological strain in tragic situations (Kuiper et al., 2014). The sense of social belonging that arises from the social aspects of humor (affiliative humor) provides a sense of partnership and collective support (Stokker, 1991). Kantar (2018) who examined letters and diaries of World War I soldiers, found that they contained diverse humor styles: self-deprecating humor, dark humor that related to the fear of death in the trenches, superiority humor that mocked the enemy, and critical humor targeting the authoritarian military establishment.

Studies have found that some humor styles, such as aggressive humor and affiliative humor, are associated with lower levels of anxiety, whereas self-deprecating humor is associated with higher levels of anxiety (Kuiper et al., 2014). Research on humor has proposed several explanations for the mechanisms through which humor reduces anxiety. Some link this to an attempt to recoup self-esteem that has been damaged in these situations, a mechanism of “self-preservation” (Frankl, 1984). Others have described a way of exerting control over uncontrollable circumstances to avoid feelings of total helplessness (Gaither, 1973; Henman, 2001). Humorous content also serves as a psychological buffer, minimizing the effect of anxiety in the context of heightened public discourse surrounding mortality (Mahat-Shamir and Kagan, 2022). Culture offers a person symbolic immortality (Lifton and Olson, 2018), and in situations of distress in which mortality becomes prominent, religious and nationalistic sentiments intensify. Collective themes are well reflected in humorous content under these circumstances (Greenberg et al., 1990, 1992).

In contemporary technology, social networks have become a key stage for humor. Humor on social media serves as a dominant agent of ideologies (Charteris-Black, 2011), leveraging the affordances of the digital space to allow for a plurality of voices, even in times of social (Milner, 2013) and political (Ross and Rivers, 2017) conflicts. During war, humor is present on all digital fronts. It is utilized as a strategic weapon, both offensively and defensively: offensively, it appears as a form of resistance to the enemy, such as through political propaganda and efforts to increase civilian engagement in the war effort (Merziger, 2007; Lyczba, 2015); defensively, it serves as a survival and adaptation tactic for soldiers (Le Naour, 2001) and civilians, even those experiencing extreme danger, atrocities, and massacres (Üngör and Verkerke, 2015). Studies focusing on the Ukraine-Russia war indicate that political leaders use social media to disseminate humorous content to boost national morale (Yehorova et al., 2023), alongside dark humor that reflects the hardships of war (Dynel, 2024).

Although extensive literature addresses the role of humor in trauma generally, a clear research gap remains regarding how specific humor styles manifest within rapidly emerging digital platforms like TikTok during active conflicts, particularly among micro-influencers and ordinary users.

### 2.3 TikTok, humor and war

Since its launch in 2017, TikTok has gradually overtaken other major social media platforms in Israel (Bezeq, 2025) and internationally (Schellewald, 2021), becoming a leading video-sharing platforms. The platform’s features – its design, diverse response formats, and the algorithm that prioritizes videos that generate interaction – encourage users to become *Tiktokers*, active creators, who are driven to create their own content rather than just consume it. These users employ a variety of strategies to enhance their visibility, leveraging trends and platform-specific features – such as hashtags, keywords, filters, and visual and audio memes – to “communicate” with the algorithm (Simpson et al., 2022). TikTok memes are often multimodal and combine layers of video, text, and sounds. However, the platform can also restrict creativity by nudging users toward specific norms of visibility (Zeng and Abidin, 2021) while “punishing” those who fail to adapt to these norms (Duffy and Meisner, 2023).

Another key characteristic of popular TikTok content is the comedic tone and the combination of acting and humor (Schellewald, 2021), which manifest in lighthearted presentations of even serious topics (Zulli et al., 2024). Indeed, TikTok is perceived primarily as a platform for entertainment content related to users’ daily lives (Abbasi et al., 2023), but many young people also consume political content on the platform (Freimann, 2024). TikTok challenges, for instance, can carry significant political undertones. Such videos serve as a platform for raising awareness about social issues, spreading ideology, and expressing political opinions through audiovisual action (Tully and Ekdale, 2014). Against this backdrop, it is easy to see how TikTok has become a dominant arena for content related to war and national conflicts in recent years. For example, the violent events between Palestinians and Israelis in 2022 were accompanied by intense activity on TikTok from users on both sides, with some even dubbing these events the “TikTok Intifada” (Divon, 2022). Similarly, during the war in Ukraine, TikTok served as a central platform for information about the conflict (Ertuna, 2023), with some referring to it as the ‘First TikTok War’ (Chayka, 2022).

Humorous content in TikTok is used, among other things, for propaganda and the dissemination of disinformation. To achieve this, users employ semi-automated tactics that combine algorithmic and human elements (Bösch and Divon, 2024). These tactics facilitate the collective processing of wartime trauma (Vickery, 2020; Cervi and Divon, 2023) and contribute to the personalization of war (Laineste et al., 2024). Research also indicates that the closer meme creators are to war zones, the more contextually detailed and culturally embedded their content. The representations adopted by content creators are influenced not only by psychological distance, but also by national media consumption patterns and cultural trends (Nissenbaum and Shifman, 2022).

Divon and Eriksson Krutrök (2023) highlight the role of war influencers in TikTok, who combine the characteristics of activist micro-celebrities and online content creators. Their use of humor to depict war events aims to achieve prominence on the platform while simultaneously increasing users’ awareness of and attention to traumatic events (Gómez, 2019). Despite the gap between everyday and war-related content and the inherent challenges in representing trauma, war influencers maintain TikTok’s distinctive language. They describe this representation as “gamified trauma,” a dynamic framework that enables creators to translate personal and collective grief, struggle, and resilience into the platform’s local and humorous language. Consequently, war events on TikTok are presented in the same language as other content on the platform, adhering to a style that fosters emotional resonance, self-reference, and user engagement (Pera and Aiello, 2024). However, some critics argue that the hardships of war undergo emotional domestication on the platform, with humorous videos reflecting an artificial sense of normality that adheres to familiar patterns, while failing to accurately portray reality. As a result, war on TikTok is presented in a tamed, sanitized, and unrealistic manner (Colombo et al., 2024).

While TikTok’s social and political roles during wartime have attracted growing scholarly attention, the intersection of war, trauma, and humor on this specific platform remains underexplored. In particular, the ways in which TikTok’s unique affordances—such as algorithmic amplification, participation in trending formats, localized audiovisual language, and a culture of remix and parody—shape how users produce and consume comedic content in the context of national crisis have yet to be thoroughly examined. These affordances raise critical questions about how humor styles and coping mechanisms are adapted to the platform’s performative, collaborative, and fast-paced logic—and, conversely, how such content may also reshape the norms and dynamics of the platform itself, particularly during periods of extreme collective distress.

Moreover, the existing literature, as reviewed above, has primarily focused on content analysis while lacking an integrative, multidimensional perspective that combines quantitative mapping of humor types and functions with the lived experiences and perspectives of the content creators themselves. Specifically, it does not sufficiently consider the creators’ motivations for producing humorous content in the context of war, the dilemmas they face, and their perceptions of how audiences engage with their content. This study aims to address this gap by offering a polyphonic lens on humor production during an ongoing national trauma, integrating systematic content analysis with in-depth interviews conducted with TikTok influencers and comedic content creators. This dual approach enables us to examine not only what is visible on the platform but also the personal, emotional, and ethical considerations that shaped these creative expressions during a prolonged crisis.

Accordingly, the research questions guiding this study are as follows:

1. What are the stylistic and functional characteristics of humorous content published on TikTok by Israeli users during the first months following the October 7th attack?
2. What are the considerations, motivations, and perceived functions underlying online influencers’ decisions to publish humorous content on TikTok during this period?
3. What dilemmas and boundaries do online influencers face when publishing humorous content?
4. How did audience interaction shape the nature of the content uploaded by online influencers?

## 3 Method

This study employs a mixed-method approach, combining quantitative content analysis of relevant TikTok videos with in-depth interviews with Israeli TikTok influencers. The analysis of videos allows us to examine the types of humor used by creators on TikTok, and characterize the humorous war-related content that was uploaded in the immediate period post October 7th, but to realize the intentions, motivations, considerations and dilemmas that accompanied creators in the creation process we need to talk with the creators themselves. Therefore, we combine content analysis with interviews with creators.

### 3.1 Participants and study corpus

For the content analysis, a sample of 257 TikTok videos posted between October 7 and December 7, 2023, was collected. We collected videos posted in the immediate 2-month term after the war embarked, as this period is seen as the first phase of the war, which ended with the release of Israeli hostages and Palestinian prisoners in a prisoner exchange deal, that included a short ceasefire before the fighting resumed at the beginning of December. This two-month period was selected because it captured the initial shock of the attack when social cohesion revolved around coping with the crisis. Videos were included if they contained humorous content related to the October 7th attack and its aftermath and were posted by Israeli creators, as identified through account information and video content.

For the interviews, seven Israeli influencers who actively posted humorous content relating to the war on TikTok during the study period were recruited for in-depth interviews. Interviewees are known TikTok creators (micro-influencers) with number of followers ranging from 50,000 to 238,000, and some are also media personas and stand-up comedians. Three of the interviewees are female and four are male. Some interviewees also served as reserve military soldiers during the research period.

### 3.2 Materials

#### 3.2.1 Content analysis

For the content analysis, a codebook was developed based on the literature describing the use and types of humor in coping with crises, trauma, and war (Pande, 2014; Ostrower, 2015; Kuiper et al., 2014), and specifically its appearance on social media (Divon, 2022; Yehorova et al., 2023; Mahat-Shamir and Kagan, 2022), as well as on themes that emerged from preliminary interviews. The codebook includes the following binary categories:

##### 3.2.1.1 Type of humor

Self-deprecating: Videos that include self-referential humor, where creators joke about their own experiences, coping mechanisms, stress, etc.

Superiority: Expressions of supremacy over the enemy (i.e., Hamas terrorists), ridiculing the enemy etc.

Parody: Imitation of genres and ridiculing recognized situations.

Satire: Combining critical messages in the content, directed outward-at Hamas terrorists or Hamas supporters, or inward at Israeli authorities or political figures.

Dark humor (Gallows humor): Jokes that may be considered tabu. Grim, or ironic humor, is specifically related to the danger of death.

Collective humor: Insiders’ jokes that use terms, slang, or experiences shared specifically by the Israeli collective. Humor is recognizable specifically by those who share the experience of war.

Community building: Use of symbols that emphasize creators’ belonging to the community (such as a flag or national symbols).

Nonsense: Simple, absurd, and non-sophisticated jokes.

Morale-boosting: Humor meant to lift the spirits of the audience.

##### 3.2.1.2 Format

Memes and trends: The use of known memes or TikTok trends in the video (songs, format).

##### 3.2.1.3 Appearance of characters in the video

Civilians, Soldiers.

##### 3.2.1.4 Content focus

Frontline: videos from the front, experiences of soldiers, the image of soldiers.

Home front: including videos showing missile attacks on Israeli cities and war-related experiences of civilians.

The categories were coded independently, and a video could feature none, one, or multiple categories.

#### 3.2.2 Interviews

A semi-structured interview protocol was developed to explore creators’ motivations, decision-making processes, and experiences. Interview questions included:

The impact of the events on interviewees’ activities on TikTok: How did interviewees react to the events, how did the war affect content creation, what functions did humor serve for them, and what were their considerations in uploading content.
Content production and boundary setting: What styles of humor did interviewees use, what boundaries did they employ, how did they approach sensitive content.
Audience reaction and personal influences: how did the audience react, how were they personally affected by the creative experiences.

### 3.3 Procedure

We conducted two interviews prior to analyzing the videos to assist in designing the codebook for content analysis. We then created a codebook and analyzed the videos. Following the quantitative content analysis, we conducted five more interviews to further investigate influencers’ experience in creating and distributing humor videos in the immediate period following the October 7 events.

The interviews were conducted via phone or video calls. To recruit interviewees, the research team personally approached them on social media or through their agencies.

All interviews were recorded, transcribed, and analyzed using Luborsky’s (1994) thematic analysis method, which is well-suited for qualitative data from semi-structured interviews (Butcher et al., 2001). This approach emphasizes the representation of participants’ perspectives and experiences. Initially, we familiarized ourselves with the transcripts by reading them thoroughly, without taking notes. We then reread the transcripts to identify and record preliminary topics. Subsequently, we grouped topics into themes. We invited interviewees to read preliminary drafts of the analysis and insights from their interviews. Essentially, we implemented the collaborative strategy suggested by Lincoln and Guba (1985), which is especially suitable for narrative research because it presumes that in social science, the perspectives of both researchers and participants are necessary to achieve a reliable and rich scientific product. Participants’ comments and feedback were carefully reviewed and incorporated into the final analysis to ensure that their perspectives were authentically represented in the study.

Videos were collected using the platform’s search tool. The search was conducted using a guest account (not logged in) on various devices over a period of 2 weeks to prevent biases based on search personalization. The data were collected using the following hashtags: “war funny,” “Israelis at war,” “Swords of Iron” (the name given to the war by Israeli government), “war,” “reserves,” “evacuees,” “hostages returned,” and “Iron Dome” (Israel’s missile defense system), as well as trending tags related to the war that appeared during the period such as “Sha-ger” (launch, in Hebrew).

From this search, 257 TikTok videos posted between 7.10.23–7.12.23 were sampled, which featured humorous content directly related to the war.

Coding was conducted by three raters trained by the researchers. Repeated training sessions were conducted until Krippendorf’s Alpha of more than 0.8 was achieved for all categories.

## 4 Results

### 4.1 Quantitative findings—videos

Table 1 summarizes the frequencies of all the coding categories.

**Table 1:** Frequency of coding categories in the videos (coding categories overlap).

Coding category			Frequency (%)
Trends/memes			64.5%
Characters			
Soldiers			39.8%
Civilians			62.9%
Video focus			
Military routine/soldier experience			27.4%
Soldier image/appearance			30.5%
Home front coping			61.8%
Types of humor			
Superiority humor			27.8%
Parody			41.7%
Satirical			11.0%
	Satire target	Hamas supporters	59.0%
		Hamas terrorists	31.8%
		Internal factors	31.8%
Self-deprecating			49.8%
	Self-deprecating focus	Coping with the events	63.0%
		Frontline experience	35.0%
Dark (gallows) humor			7.7%
Collective humor			45.2%
Humor functions			
Morale boosting			75%
National and community building			10.8%
Nonsense			29.3%

#### 4.1.1 Format

The analysis reveals that a large majority of the videos follow familiar trends or used memes. They contained popular catchphrases, formats, and characteristics. Some were general, familiar TikTok trends (e.g., remakes of Disney musicals), and others featured trends related to the war (e.g., sounds taken from the Air Force command). These videos follow platform-specific formatting characteristics and creative practices.

#### 4.1.2 Characters in the videos

Videos featuring civilians were more dominant, but no less than 40% of the videos featured soldiers, suggesting soldiers’ need to engage with the events through humorous expressions on the popular platform.

The majority of the videos focused on the home front’s coping with the war (e.g., running to shelter, mimicking news anchors). Videos focused on military routine and soldiers’ experience in the front (e.g., soldiers dancing in assembly areas), were similarly common as videos focused on the image, the appearance and behavior of soldiers (e.g., growing moustache trend among soldiers).

An analysis of the timeline of the videos found that videos by civilians were prominent at the beginning of the research period, while videos featuring soldiers and focusing on the war front became more dominant later. In this context, it is important to note that during the weeks following the attack, operational activities were limited, and the main sentiment in Israeli media and public discourse revolved around processing the events of October 7.

#### 4.1.3 Types of humor

The data show that superiority humor (e.g., ridiculing Hamas leaders) was less common than other types of humor. In contrast, parody (e.g., editing a children’s shows by replacing the face of the hero with the face of a civilian who fought Hamas terrorists) appeared at a relatively high rate. The timeline shows a decrease in superiority humor and parodies over time. Collective humor, which expresses shared experiences and emphasizes a sense of shared identity, was dominant (for example, soldiers singing a familiar moral song during a visit by the chief of staff), as was self-deprecating humor, in which content creators reflected themselves, their social group, or their community at the center of the joke. Most self-deprecating videos deal with the nature of coping with the events, whereas others revolve around the frontline experience of war. In contrast, dark (gallows) humor was quite rare. Satirical videos appeared at a particularly low rate – in 11% of all videos, of which the most common criticism was directed at Hamas supporters, and satire directed towards Hamas terrorists or internal factors was rare.

The data indicate that content creators tended to combine a variety of types of humor, with superiority humor and satire being less common, while self-deprecating and collective humor appeared at a higher rate. Dark humor, dealing directly with the trauma and risk of death, was relatively rare, perhaps indicating the caution exercised by creators who preferred to refrain from dealing with the most sensitive parts yet.

#### 4.1.4 Humor functions

The findings indicate that one of the main functions of the videos was morale boosting, while elements of national and community building were not very common. Almost a third of the videos documented moments of meaningless nonsense humor.

#### 4.1.5 Classes of humorous videos

To characterize the main profiles of humorous content that surfaced on TikTok during the study period, a Latent Class Analysis (LCA) was performed. LCA is used to characterize a multidimensional discrete latent variable based on a cross-classification of multiple observed categorical variables, by thus providing an overview of profiles (classes) of observed variables based on shared characteristics. The analysis was conducted in R with the poLCA package (Linzer and Lewis, 2011). We ran models ranging from 2 to 6 classes, set for 5,000 Iterations to ensure global convergence. The best model selection was based on the Bayesian Information Criterion (BIC) value, which was lowest for four groups (BIC = 3442.99; AIC = 3233.14), combined with examination and interpretation of the model and its practical usability.

The analysis found that the videos were split between the four classes similarly, with the classes containing civilian videos being slightly larger.

Table 2 summarizes the main characteristics of each class.

**Table 2:** Characteristics of classes found in LCA.

Category	Class 1 (28.4%)	Class 2 (21.8%)	Class 3 (28.8%)	Class 4 (21%)
Dominant participants	Civilians (1.00)	Soldiers (0.95)	Civilians (1.00)	Soldiers (0.83)
Typical setting/topic	Home-front life (1.00) and coping (0.93)	Frontline (0.71), soldier image (0.71), war routine (0.67)	Home-front matters (0.98)	Frontline routine (0.58); soldier image (0.54)
Self-deprecating humor	**Highest** (1.00)	High (0.98)	Absent (0)	Absent (0)
Collective/group humor	**Moderate** (0.54)	Moderate (0.43)	Moderate (0.34)	**Moderate** (0.51)
Parody	Moderate (0.43)	Moderate (0.32)	**Highest** (0.61)	Low (0.24)
Satire	Very low (0.08)	Very low (0.05)	**Low, highest among classes** (0.20)	Very low (0.07)
Superiority humor	Very low (0.15)	Very low (0.12)	**Highest** (0.44)	Moderate (0.38)
Dark/black humor	**Very low, highest among classes** (0.15)	Very low (0.05)	Very low (0.08)	Absent (0)
Morale-boosting	High (0.70)	High (0.80)	High, lowest among classes (0.66)	**Highest** (0.87)
Featuring soldiers	Rarely (0.04)	Nearly always (0.95)	Never (0)	Very common (0.83)
Featuring civilians	Always (1.00)	Rarely (0.09)	Always (1.00)	Unlikely (0.18)
Frontline imagery/content	Rare (0.08)	Common (0.71)	Almost never (0.02)	Moderate (0.58)
Home-front imagery/content	Always (1.00)	Rare (0.05)	Certain (0.98)	Unlikely (0.18)

Probabilities appear in parentheses. Main characteristics of each class appear in bold.

Class 1 (28.4% of the videos): Self-deprecating humor about coping, civilians joking at the home front: This class featured civilian-focused self-deprecating humor focused on the home front, with content covering coping with the situation, combining morale boosting and some collective humor (pr. = 0.54). Following Ostrower (2015) and Stokker (1991) these videos provide therapeutic functions that assist in interpreting the threats and strengthen sense of collective support. These videos were very *unlikely* to feature soldiers or any frontline-related content. Also, these videos were highly unlikely to feature satire, superiority or dark humor.

An example for videos in this class can be seen in the video posted on October 13, titled: “Preparation for war.” The creator is featured with her mother at the supermarket. The video is accompanied by the caption “Nobody: My mother: loots the supermarket after a Home Front Command announcement.” The video is accompanied by a viral recording of the Prime Minister announcing a national state of emergency. The video ridicules the exaggerated preparations and “looting” of groceries by civilians on the home front preparing for the war.

Class 2 (21.8%): Self-deprecating humor from the frontline, soldiers mocking war routine and the soldier image: This class featured self-deprecating humor, often related to the frontline, focused on the image of soldiers or the war routine. These videos almost always featured soldiers and aimed to boost morale. Similarly to class 1, these videos offer therapeutic functions to interpret and process the situation-among soldiers. These videos were highly *unlikely* to feature civilians, contain home front topics, satirical elements, black or superiority humor.

An example for videos in this class can be seen in the video posted on October 12, showing a female soldier marching and carrying enormous combat gear, stops to rest on a rock and falls backwards. The video mocks the image of frontline combat soldiers.

Class 3 (28.8%): Parodies: home front civilian-focused videos. This class contained only civilian videos that mostly dealt with topics related to the home front. Many of the videos featured parodies and collective humor. This category had the highest probability of superiority humor and slightly more likely to include satire compared to the other classes. This class had no self-deprecating humor and was very unlikely to have dark humor. Additionally, it had the lowest probability among the classes to feature morale-boosting videos. Such content may reflect its creators’ lower levels of anxiety, which enabled them to produce more aggressive content (Kuiper et al., 2014) and be less occupied with morale-boosting and collective spirit-lifting.

An example for this class can be seen in the video posted on October 26. The video shows a young man imitating a very familiar TV reporter. The man hides under a table, reporting an ongoing missile attack from which he is hiding. This is a parody based on an actual scene that occurred when, in the middle of a live broadcast, an alarm was sounded and the reporter continued broadcasting under cover.

Class 4 (21%): Collective humor to boost morale—soldier-focused: This category had a moderate probability of collective humor and a high probability of morale-boosting content. The videos were mostly soldier-focused and often related to the military routine at the frontline or the soldier image. There was no self-deprecating humor or dark humor, and a low probability of parodies or civilian-focused content. These videos mainly emphasize a sense of belonging, unity, and suggest soldiers’ efforts to corroborate and strengthen themselves and their brothers-in-arms in the face of war.

An example is a video showing several soldiers shaving and leaving their mustaches. The mustache trend began at the beginning of the war, in which conscript and reserve soldiers documented themselves with mustaches. The mustache is a sign of shared destiny between the soldiers, and some see it as a tribute to the fighters of the Yom Kippur War of 1973.^2^ The song “Getting out of depression,” which has gone viral and represents Israelis’ optimism, accompanies the video. The video is also accompanied by the caption: “Joining the trend with all our might.” This video, and the mustache trend in general, illustrate how soldiers use creative practices to link contemporary experiences to collective memories of past confrontations, forging a connecting thread between past and present national traditions of resilience in the face of danger.

### 4.2 Qualitative findings—interviews

Participants described how immediately following the October 7th attack, the events were so overwhelming that they found themselves in a state of shock and fear, making it difficult for them to engage in humor: “When the attack happened and the war began, during those first few days, I felt like I was in shock. I could not respond—I was completely frozen” [Yoav].

Only after the initial shock could participants engage in humor and upload comedic content.

#### 4.2.1 Motivations to use humor

Analysis of interviewees’ words revealed that humor was employed in two distinct ways. On the one hand, it served as a defense mechanism, helping participants and their followers escape the highly stressful situation. On the other hand, humor was used as a weapon to combat Hamas terrorists and their supporters worldwide, addressing what they perceived as misconceptions about the war.

##### 4.2.1.1 Humor as a defense mechanism

Participants described employing humor as a defense mechanism, serving multiple psychological functions. Humor was utilized as a form of self-therapy, offering emotional relief and facilitating coping; as a means of escapism, allowing temporary detachment from distressing realities; and as a reflective mechanism, enabling participants to process their experiences with greater emotional distance. Additionally, self-deprecating humor emerged as a distinct strategy, functioning as a protective mechanism against vulnerability and external judgment.

###### 4.2.1.1.1 Humor as self-therapy

After the initial shock of the October 7th attack, participants felt that humor became their way to find a sense of hope, optimism, and normalcy amidst the horrific situation. It allowed them to detach from the chaotic and tragic world around them.

“I realized that I needed humor because one cannot continue to endure all the tragedies without it. Humor is the only thing that helps you feel normal again and provides a sense of optimism. When you laugh at something, it allows you to view it from a bit of a distance. That’s the beauty of humor; it helps you step back from the trauma and chaos. It also offers a sense of relief, instilling the belief that 1 day we can laugh about it even more. I mean, we will get through this, and it brings a lot of hope” [Noga].

They described humor as a defense mechanism, with one participant stating, “I think it was Charlie Chaplin who said, ‘Life is a tragedy in close-up, but a comedy in long-shot.’ For me, humor was a way to zoom out; it was and is my defense mechanism” [Ido].

###### 4.2.1.1.2 Humor as escapism

Humor can be viewed as a form of escapism, where individuals avoid directly confronting stress-inducing problems while striving to maintain a positive outlook. This aligns with the concept of hedonistic escapism as described by Stanisławski (2019), which includes humor-based coping strategies. In the context of the unpredictable and uncontrollable events of the October 7th attack and the subsequent war, participants found that using an avoidant yet positive strategy, such as humor coping, was an effective way to manage their stress. They actively sought out humorous content to watch.

“In the first few days, I had nearly 24 h of screen time because I could not sleep. I kept searching for something funny, as humor was my way of coping with everything that was happening. I was actively seeking laughter, almost desperate for it. It was too hard to focus on reality and what was unfolding, so I escaped into my phone and found solace in humor” [Avichai].

Participants believed that humor served not only as their means of escaping the horrific reality but also to help their followers engage in escapism amid the highly stressful situation.

“From the reactions of my followers, I realized that there are people eagerly waiting for my humorous content. Those who, like me, were struggling and feeling alone, could benefit from a funny video that reminds them they are not alone. My goal was for the audience to watch and find a space to disconnect” [Yoav].

###### 4.2.1.1.3 Humor as a reflective mechanism

Participants tapped into their own humor and started uploading comedic content to express their feelings and normalize their fears. Some videos used humor to depict the situation itself, portraying the fear caused by the attack and normalizing these fears.

“Everyone was afraid of everything, and people were openly discussing their fears; even TV presenters were crying. It felt like emotions were so visible that I suddenly felt normal, realizing, ‘Wow, everyone is struggling like me.’ I did not have to hide it. In one video, for example, I played sad piano music while joking about fears. I mentioned how my old fears have vanished. Now, when I see a cockroach, I say, ‘Come on, sit down, watch TV with me’” [Hadar].

###### 4.2.1.1.4 Self-deprecating humor

Participants used humor to defend themselves from the stressful war situation by laughing at themselves and their circumstances, even finding humor in their efforts to protect themselves and those around them, such as their children.

“The first video I uploaded was about how I mediate the war for my children. I pretended to save water, telling them it was due to global warming, and when we went to the shelter, I said we were going to discuss things with the house committee. I spoke to the other neighbors in the shelter in English, and then, my daughter mentioned that she understood English, I suddenly switched to speaking in Chinese” [Noga].

Participants used humor both to protect themselves and to offer a sense of defense to their followers. At the same time, humor was wielded as a weapon against Hamas terrorists and their supporters worldwide. This will be illustrated in the following theme.

##### 4.2.1.2 Humor as a weapon

While all participants stated that they generally avoid humor that could hurt others: “I will never upload videos making a mockery of someone, videos that may hurt someone” [Avichai], they all used humor as a weapon against Hamas terrorists and their supporters around the world.

“I created satirical videos opposing support for Hamas. For example, I posted one with my sister where we mimicked college-style videos, with Israel portrayed as the only girl who can see the truth, while everyone else remains oblivious—just like in a movie where the audience recognizes the bad character, but the rest of the cast does not. Humor makes it easier to reveal the truth because a comedic twist can make it unique and funny. I believe people have come to accept a lie without really investigating. That’s what I love about comedy: you can tell the truth in a funny and bold way without coming across as aggressive” [Yoav].

In light of their frustration and anger over the situation and what they conceive as the world’s lack of empathy toward Israel’s suffering, they chose to address an international audience by uploading videos in English.

“I uploaded videos in English. I wanted to highlight the absurdity of the world because I was shocked by it too. I’ve spent my life fighting for women and against sexual crimes, and suddenly, you see the #MeToo movement is not with us. It felt absurd like the whole world was reaching a point of absurdity, and I felt it was something that needed to be fought against” [Hadar].

Ridicule has long been seen as a powerful weapon, often reserved for extreme situations. In Christianity, ridicule is generally viewed as sinful, except during war (Waller, 2006). In the Talmud, mockery is forbidden except against idol worship, reflecting its destructive power (Cohen, 2006). Early in Islam, Muhammad used ridicule as a tool of war, with poets crafting satirical attacks on enemies (Canadian Society of Muslims, n.d.). Thus, during the war against Hamas, participants used ridicule and humor as a weapon against Hamas and its supporters.

“The act of laughing carries a lot of meaning; some people may interpret it as contempt, but it is not. Laughing is a way of letting go, and when you laugh, you feel victorious. When you are scared, you cannot laugh because fear holds you back. But as soon as you start laughing, it’s a sign that you have moved past the fear stage, and now you can truly begin to win. So, I started uploading videos of fake phone calls in English. For example, when it was reported that Susan Sarandon said the rapes of women were “part of the context” and her agent fired her, I uploaded a video where I played her agent, calling her to fire her, and telling her she’s a great actress but just stupid. It really took off. After that, I started making similar calls to others in the headlines—Sinwar, the Red Cross, Roger Waters, the UN, Hamas, Starbucks, Harvard, and its president. Whenever there was a big news story, I quickly pretended to call them, and it was highly satirical. It felt amazing like I was confronting a common enemy” [Hadar].

Recognizing humor as a powerful weapon, participants described carefully checking and double-checking themselves before using it: “I tried to be very thoughtful and cautious with what I uploaded, asking myself countless questions-probably 50 questions, 50 thousand times-before posting a video.” [Ido]. In addition to carefully checking themselves, participants sometimes deleted videos when they felt the weapon of humor might backfire.

#### 4.2.2 Dilemmas and boundary setting

The participants were greatly occupied with what is and is not appropriate in posting humorous content immediately after October 7th:“I try as little as possible to touch places that can hurt people. I do not want to do it online because it can reach so many people and I do not want anyone to get hurt” [Yoav]. The perception that the imaginary audience includes a variety of people in different situations requires them to be very cautious. From the analysis of the interviews, it is possible to outline several layers of boundaries that participants set for themselves, some of which resulted from negotiations with their audience.

##### 4.2.2.1 A boundary of timing

An observance of the quantitative content analysis and the analysis of the interviews reveals that there may be a difference between the timing of humorous videos posted by common users compared to TikTok influencers. While many users uploaded humorous videos immediately, in the first days following the events, the interviewees, all influencers with high profile accounts, said that they have not seen fit to post humorous content for a long period after the events. Although they began posting on TikTok immediately, they postponed the humor content for a while: “Of course, we stopped with sketches and advertising content and whatever, and we just did “Hasbara” [promoting Israeli narrative abroad] … I think I posted other content than hasbara only after about a month and a half” [Noya].

A similar differentiation between mission activities and entertainment activities is illustrated in Hadar’s words: “At first, I really avoided [entertainment content]. I really avoided it. What I did was I was in a frenzy of uploading photos of missing persons, and that’s what I saw that all my colleagues started doing as well. Certainly not to joke and certainly not to laugh.”

The legitimacy to engage with humor is not set at a single point in time, but an ongoing negotiation that is also related to the context of the video. For example, Ido describes that even after he began posting humorous videos, there were some cases in which he felt it was still too early to post a video. He describes recording a TikTok video with a famous influencer, during which an alarm went off. They recorded the event and published it:

We posted the video and named it: “In the Safe Room with Noa Bog” [the influencer’s name], it went really well but I felt bad in retrospect that there was a siren, as if it was an exploitation of the situation and it still felt too early to me.

##### 4.2.2.2 A boundary of content: potential harm to the recipients and the objects of humor

The perception of the differential sensitivity of the imagined audience led participants to consider the potential harm of the vulnerable among the recipients, indicating a form of therapeutic intuition and heightened emotional sensitivity to their audience’s psychological states:

“When there were abducted children in Gaza, I could not create contents that complain about my children. I will not laugh at, let us say, if I had a punch line about the public kindergarten, you are picking up the kids, it’s like a child in captivity, you cannot make captivity jokes anymore, prison jokes, you cannot have all these things that have to do with something that’s a bleeding wound. It is impossible, in any way. I cannot laugh at what it’s like to be a widow, I cannot laugh at what I’m not, God forbid I will not be either, but I can only laugh at the things I experience through my eyes, with a very high sensitivity to the people who are suffering around me. I cannot disconnect from it. And I do not want to either. Of course, I want to emphasize the absurdity of the injustice done to people, not the trauma that people are experiencing” [Noga].

This caution is not only regarding the humorous content, but also regarding the choice of words. Ido described how he carefully examined every word in the text, in order to make sure that the text did not contain words that might trigger his audience, demonstrating an intuitive understanding of trauma-informed communication (van der Kolk, 2014):

“At first, I wrote the word ‘at the funeral’ in the sketch, and then I wrestled with myself with this word. Is this a trigger? Is it okay? And I dwell for an hour only on the question of whether to replace the word ‘funeral’ with the word ‘Shiva’a’ or ‘memorial’.”

From another direction, Noya relates to her refraining from producing humorous content about a specific person or story: “I think I’ll laugh at almost everything. But I will not laugh at someone else’s specific personal story, because it’s not my place.”

Additionally, participants expressed solidarity with Israeli society as another dimension of their therapeutic sensitivity. By intentionally choosing not only what humor to omit but also what humor to create, they aimed to foster a sense of community resilience and collective emotional support during the ongoing conflict (Bleich et al., 2003; Mancini, 2019). Thus, humorous content served not merely as entertainment but also as a form of emotional affirmation and social solidarity, enhancing the therapeutic potential inherent in digital communication during traumatic public events (Naslund et al., 2020).

##### 4.2.2.3 A boundary of content: the style of humor

The decision regarding the appropriate and inappropriate style of humor is a matter of dispute among the participants. Thus, there were those who defined black humor as “off-limits” due to the difficult events:

“In real life, one-on-one, I like all kinds of humor, I do not care, but in my videos I try as little as possible to touch things that can hurt people… So now black humor on the Internet is not… It can reach so many people and I do not want anyone to get hurt. I want them to laugh and have a good atmosphere. Because life is hard and heavy as it is” [Yoav].

Yoav points to the demarcation of the line between what is permitted “in real life” and what is forbidden in the online space. Ido, on the other hand, rejects this division:

“In performances, I like to go for black humor. I also like black humor on the Internet, and I love the fact that half the world is triggered, and half of the people explode with laughter more than with any other type of humor. And I think it’s also the most healing humor somehow, so specifically the things that are a little more painful. And I tried to just do this kind of humor, because that’s the humor that people recognize of me and it’s the kind of humor that I know how to convey a lot with, what I feel in the end. As far as I’m concerned, I’ve always thought that it’s okay to laugh at everything, but what I’ve understood from the past year on the Internet is that it’s okay to laugh at everything as long as it’s funny” [Ido].

Later in his remarks, he defines walking on the edge as something deliberate and preferred by him:

“Something in me likes to flirt with the border. With the limit of when it will be too much for people. And people will always have something to write, so especially during the war […] the reality was extreme enough for the content about it to be extreme… for me, the threshold will always be: if it makes me laugh, then it will probably make other people laugh” [ido].

##### 4.2.2.4 A boundary of contextual implications: damage to the war effort and the image of the state

The sense of collectivism and mobilization was expressed not only in the style of humorous content, as illustrated above, but also in boundaries. For example, Alex describes a case in which he uploaded a humorous video against the backdrop of the war in Gaza, but after he raised concerns that “the video could spread and go viral.” he decided to delete it for fear that it would damage the country’s image.

“There were videos I uploaded and then deleted to avoid backlash on the global stage. For instance, there’s a trend where someone is asked, ‘Where do you live?’ and they respond, ‘In this apartment,’ followed by a question about their rent, which leads to revealing a rundown place. I filmed one inside Gaza that was both funny and heartbreaking. After about half an hour, I deleted it because I realized it could go viral worldwide. I did not want our country to face backlash because of it, so I removed that content” [Alex].

This response reflects the responsibility and awareness shown by Israeli content creators for the explosiveness of the platform on which they upload videos.

## 5 Discussion

Psychological research on well-being and quality of life has traditionally emphasized the impact of negative factors, such as adverse life stressors like the October 7th attack, which contribute to reduced well-being or the onset of psychopathology (Mahat-Shamir et al., 2018a; Mahat-Shamir et al., 2018b; Mahat-Shamir et al., 2023). However, over the past two decades, an alternative perspective rooted in developmental psychology has emerged, focusing on the personal, environmental, and contextual factors that enhance well-being, particularly under adversity or stress (Windle, 2011; Zautra et al., 2008). This perspective, termed resilience, is defined as “the process of effectively negotiating, adapting to, or managing significant sources of stress or trauma, facilitated by assets and resources within the individual, their life, and environment” (Windle, 2011, p. 153). Resilience has been studied across the lifespan using a multidisciplinary approach. Few resilience researchers have identified a sense of humor as a distinct protective factor in resilience. However, humor researchers have long acknowledged its value as a coping mechanism for navigating life’s challenges (see for example: Abbasi, 2017; Cherry et al., 2018).

This study examined the role of humor in building resilience in a major national trauma event, and the role of TikTok as a creative platform enabling creators to communicate through humor with the local audience, international audiences, and above all-with themselves. It offers a unique contribution to the growing body of scholarship on digital humor in times of crisis by examining not only the sociological functions of humor during a national trauma, but also the role of platform-specific structures in shaping the ways humor is produced, framed, and circulated. Rather than treating TikTok as a neutral channel, we highlight how its affordances (algorithmic amplification, trend-based formatting, and participatory audiovisual culture) condition and constrain humorous expression, particularly under the heightened emotional climate of war and public trauma. In doing so, the study situates humor as a form of platformed performance, deeply entangled with the logic and limitations of the medium through which it is expressed.

A further contribution of this study lies in its integrative methodological design. By combining a large-scale quantitative content analysis of 257 humorous TikTok videos with in-depth qualitative interviews with content creators, we provide a multi-layered, polyphonic perspective that addresses both *what* was published and *why* and *how* it was created. This dual lens enables a richer understanding of the psychological, social, and ethical considerations involved in humor production during crisis. Importantly, the study was conducted in close temporal proximity to the traumatic events of October 7th, allowing us to capture reflections, motivations, and dilemmas as they unfolded, rather than retrospectively. The interviews reveal not only creators’ efforts to lift morale or entertain, but also their intense negotiations around timing, tone, audience sensitivity, and moral responsibility.

### 5.1 Humorous expression on TikTok as a response to public and personal trauma

The primary and significant conclusion of this study highlights the fascinating-albeit well-known-phenomenon of individuals using humor even in the immediate aftermath of, and during, severe personal and collective trauma (Kuiper et al., 2014; Pande, 2014). Moreover, this study adds a novel dimension to this phenomenon by demonstrating that this function of humor is not confined to interpersonal dynamics (Ostrower, 2015) or mediated yet limited forms of communication (Kantar, 2018). Instead, it extends to a broad array of humorous expressions on a public, interactive, and multifaceted platform like TikTok, involving diverse audiences and meanings. This platform facilitated humor both through ordinary users and prominent social media influencers, emphasizing its role in resilience-building and coping in times of extreme collective distress.

The findings indicate that the humorous content tends to follow the rules of the platform: Trends, TikTok formats and Parodies were quite dominant, suggesting that while everything was not normal for the creators, they still used familiar platform practices and formats. During the immediate period following the trauma, creators mostly aimed for lifting the spirits of their followers and themselves by posting morale boosting content and avoiding controversial and hurtful topics. Profiling classes of humor content reveals that civilians and soldiers used humor differently in their videos: When civilians offer self-deprecating content, it revolves around coping, getting ready, stressing out from the war at the home-front. Soldiers also used self-deprecating humor, but focused on the frontline and the army routine. This seemingly expected distinction is not necessarily obvious: the great majority of civilians in Israel were once soldiers, are highly familiar with the army routine and every Israeli has close relatives that were recruited for the frontlines. Therefore, there is quite little real distinction between soldiers and civilians in Israeli society. Still, the content differs significantly.

Additionally, videos featuring parodies, satire, and superiority humor were mostly posted by civilians. Civilians also were more likely to offer collective humor to reinforce sense of belonging, pride and social cohesion, sometimes at the expense of an “other” who is mocked, criticized or ridiculed. The low rate of satirical videos is interesting when considering that the mainstream media did engage quite a lot with criticism of international supporters of Hamas, anti-Israel sentiments, but most of all-inward criticism aimed mostly at the government for not preventing the horrible massacre and not functioning at the time or the events (Karniel, 2024). Still, creators felt that TikTok was not the right platform for pungent satire and the time was not right for criticism, especially one directed inward. This type of content did not belong within the rules and boundaries of the platform.

### 5.2 Political silence and therapeutic presence: navigating crisis through humor

The overall characteristics of humorous manifestations, along with the relative absence of political content, indicate a significant departure from how the platform’s political affordances have been characterized in prior research. Previous studies have highlighted TikTok’s capacity for ideological expression and institutional critique, especially through creative formats such as remix, audiovisual parody, and trend-based engagement (Literat and Kligler-Vilenchik, 2023). Other works have demonstrated how these formats can subvert dominant narratives and facilitate media criticism (Literat et al., 2022), and how algorithmic amplification may at times even support, rather than suppress, political or activist content (Grandinetti and Bruinsma, 2023). Despite this potential, it appears that during the initial stages of the October 7th war, such expressive capacities were largely left untapped when it came to inward-facing political satire or critique. As noted above, the quantitative analysis indicates a clear absence of overt political content directed at domestic leadership. The few instances of satire were primarily targeted at external enemies rather than internal institutions or figures.

This gap can be understood in light of the creators’ own accounts, which highlight several interrelated factors contributing to their self-restraint. For instance, the sensitive presence of bereaved families and hostages on the platform created a moral atmosphere in which humorous or satirical content—even if legitimate—risked being perceived as offensive or inappropriate. On a deeper level, the traumatic nature of the events—marked by extreme brutality and mass civilian casualties—triggered a heightened sense of existential anxiety and led to an urgent collective need for national solidarity and resilience. Within this emotionally charged context, political satire was often seen as inappropriate and divisive.

Indeed, when mortality is salient, people seek symbols that reaffirm a shared worldview (Greenberg et al., 1990, 1992). The TikTok videos produced by Israeli creators follow this pattern, emphasizing a unified Israeli identity and collective solidarity. In doing so, they nurtured community resilience, emotional cohesion, and morale boosting, offering a therapeutic message to fellow Israelis coping with the horrors and uncertainty of war (Bleich et al., 2003; Mancini, 2019). By refraining from satire and inward-facing criticism that may fracture that shared identity and perhaps weaken the sense of solidarity, TikTok creators appeared to curate a “we-code” of humor, focused on morale-boosting and benign self-deprecation. This restraint, we argue, is in itself a form of collective defense mechanism: it shores up a valued social self precisely when that self is threatened, reduces existential anxiety, and in turn facilitates psychological recovery. Our findings thus extend existing humor scholarship by showing that not only the presence of affiliative jokes but also the deliberate absence of internal satire and dark humor can foster communal resilience in digital spaces during the early phases of an ongoing war.

From the creators’ perspective, TikTok was not experienced as a platform detrimental to users’ well-being (Kagan et al., 2025; McCrae et al., 2017; Shan et al., 2021), but rather as a therapeutic space. While adhering to platform conventions, creators employed humor as a form of self-therapy, soothing their own wounds while simultaneously recognizing that their content also offered comfort, strength, and a sense of belonging to their followers. The act of creating and posting content became a method of trauma processing: by articulating their experiences through humor, they not only navigated their own emotional pain but also cultivated a communal space of shared solace. As they became more aware of their significance for others in distress, many came to see their content as a form of moral support—meant to uplift spirits and provide emotional escape for their immediate and proximate audiences. As awareness of TikTok’s international reach grew, some creators deliberately adapted their messages, crafting humorous yet persuasive content aimed at global viewers.

This decision to target international audiences corresponds with a wide public This decision to target international audiences corresponds with a wide public debate in Israel, criticizing the Israeli public diplomacy (“Hasbara”) in being too weak, responsive rather than reactive, focused on explaining rather than influencing opinions, and actively losing the war on public opinion.^3^ However, this creative process was far from spontaneous; it was highly calculated, marked by uncertainty, and heightened emotional sensitivity. In negotiating these complexities, participants actively engaged in resilience-building practices. Writing and producing content thus functioned not only as personal coping mechanisms but also as acts of communal caregiving. For both the creators and their audiences, humor became a weapon against despair and a fragile but vital lifeline amid unimaginable horror.

In other words, political expression on TikTok is shaped not only by the platform’s technical and algorithmic infrastructure (Grandinetti and Bruinsma, 2023), but also by historically contingent sociopolitical dynamics, evolving communal norms, and affective atmospheres that emerge in moments of collective crisis.

### 5.3 Ethical boundaries and collective taste of humor during times of trauma

The sensitivity of the moment and the effort to avoid causing harm led content creators to draw layered boundaries around the use of humor during the traumatic period of war. Some of these boundaries were based on personal judgment and emotional assessment, while others emerged through implicit negotiations with the audience. These boundaries concerned multiple dimensions: the timing of humor and its proximity to the traumatic events; the target of the joke and its perceived appropriateness; the style or tone of humor employed; and the contextual implications, such as potentially undermining the national war effort or upsetting bereaved families. In drawing these moral lines, creators sought to resolve the social and ethical tensions associated with humorous expression amid national grief, uncertainty, and collective vulnerability.

The restraint around political satire and boundary-pushing humor exemplifies how communities construct symbolic boundaries and collective taste regimes—normative distinctions about what is considered morally appropriate or inappropriate. These boundaries tend to sharpen during periods of crisis, when collective norms converge around values such as solidarity, respect, and moral duty (Bourdieu, 1984; Lamont, 1992). The decisions made by content creators in our study reflect such boundary work and are especially notable for their deliberate and self-reflexive character.

This dynamic also resonates with the Benign Violation Theory (McGraw and Warren, 2010), which suggests that humor arising from violation, such as tragedy or transgression, is acceptable only when perceived as non-threatening, typically requiring a certain degree of psychological distance from the violation. While our findings affirm that creators actively negotiated forms of distance (temporal, emotional, and symbolic), the presence of humorous content mere days after the traumatic events complicates the stricter interpretations of this theory. It may suggest that psychological distance is not the sole moderator of humor’s acceptability; rather, its reception is also shaped by the perceived function of the humor (e.g., support vs. critique), the intended audience, and the collective emotional climate. In other words, the boundaries of legitimate humor in times of crisis are not only psychological but also sociocultural, defined through symbolic distinctions, emotional atmospheres, and evolving community norms.

## 6 Limitations and future research directions

In this study, we interviewed a small sample of seven influencers to explore creators’ motivations, decisions and experiences, and complement our analysis of the videos. We acknowledge that this sample may not be regarded as representative of Israeli content creators who engaged with the war by posting humorous content. Future research could extend the examination of influencers’ perspectives by including larger, more diverse samples of influencers. Research could focus on the dynamics of humor in interactive digital platforms during crises by examining a broader range of conflicts, crises (e.g., natural disasters) and cultural contexts (e.g., different regions or cultures). Additionally, it is essential to explore the gendered perspective on the use and dissemination of humor on TikTok during collective trauma, given the well-documented gender differences in humor usage (Hofmann et al., 2023), as well as a focused inquiry into the mechanisms of legitimation (Van Leeuwen, 2007; Zeeuw, 2020) adopted by social media influencers to justify the use of humor in such contexts. As this research focused primarily on the creators and the content they created, an examination of the perceptions and response of the audience, TikTok or social media users who consumed war-related content, can offer insights into their desired content and how they were emotionally and psychologically affected by humorous content they consumed on these platforms.

## Data Availability

The original contributions presented in the study are included in the article/supplementary material, further inquiries can be directed to the corresponding author.

## Footnotes

^1^ https://govextra.gov.il/mda/october-7-massacre/october-7-massacre/october-7th-hamas-invasion-and-atrocity/

^2^ https://www.timesofisrael.com/for-reservists-growing-a-mustache-is-a-symbol-of-this-war/

^3^ https://www.inss.org.il/publication/hasbara/
